# The new Foundation Programme Mental Health Curriculum: foundation doctors’ perceptions of its importance and their competency: pre–post psychiatric placement evaluation study

**DOI:** 10.1192/bjb.2024.18

**Published:** 2025-06

**Authors:** Ioana Varvari, Thomas Dewhurst, Corinne Jones, Richard Haslam

**Affiliations:** 1 Oxford Health NHS Trust, Oxford, UK; 2 South London and Maudsley Mental Health NHS Trust, London, UK

**Keywords:** Postgraduate medical education, foundation doctors, curriculum, competency, psychiatric placement

## Abstract

**Aims and method:**

The new 2021 UK Foundation Programme Curriculum mandates foundation doctors to acquire mental health competencies. This study aimed to evaluate the effectiveness of psychiatry placements in facilitating competency attainment, foundation doctors’ perceived importance of acquiring these and their preferred teaching methods. Utilising Kirkpatrick's evaluation framework, the study employed a pre–post intervention design assessing the impact of psychiatry placements on 135 foundation doctors across three cohorts from August 2021 to March 2022.

**Results:**

Initially, foundation doctors assigned high importance to mental health competencies. Post-placements, this perceived importance improved slightly, whereas that of clinical skills scenarios slightly decreased. Significant confidence increases were observed in recognising and assessing specific psychiatric disorders. Foundation doctors favoured small seminar groups and on-the-job *ad hoc* teaching. Qualitative insights underscored the need for context-specific teaching.

**Clinical implications:**

Psychiatry placements enhance foundation doctors’ confidence and perceived importance of mental health competencies as specified by the curriculum. Addressing clinical scenario gaps through context-specific teaching and transferable skills development is essential. Customised teaching approaches, especially small seminars and *ad hoc* teaching, hold promise for effective mental health training.

The UK Foundation Programme, established in 2005, is a 2-year postgraduate training programme for newly qualified doctors that provides a comprehensive framework for the development of clinical and professional competency through a series of 4-month-long placements.^1^ Historically, foundation doctors were placed predominantly in acute trusts across a wide range of medical and surgical specialties; however, owing to the evolving medical education landscape and recognition of the importance of mental health competencies, the Foundation Programme was broadened to include optional psychiatry placements, with approximately 45% of all foundation doctors being expected to be placed in psychiatry.^2^ Mental health competencies were not made compulsory at that time.

More recently, mental illness has been recognised as one of the top ten leading causes of burden worldwide, with overwhelming pressure on healthcare providers to meet population needs.^3^ Improving training in psychiatry competencies for healthcare professionals in various settings is necessary to manage the impact of this ‘mental health pandemic’. Consequently, Health Education England updated the UK Foundation Programme Curriculum (UKFPC) in 2021,^4^ mandating the acquisition of mental health knowledge and skills under areas of core learning (Box 1).

Box 1 Mental health conditions that foundation doctors should be able to recognise and assess, and skills they should be able to apply to clinical scenarios.

**Table:** 

Mental health conditions	Clinical skills
Depression Mania Psychosis Anxiety/panic Personality disorder Delirium Chronic cognitive impairment or dementia Eating disorders Addictions Somatisation disorders and functional syndromes	Mental health legislation Ethical frameworks for difficult decision-making Interplay between physical disease and psychiatric symptoms Serious adverse effects of psychopharmacology Management of challenging patients at risk

How do foundation doctors perceive the importance of acquiring psychiatric skills at this training stage, and can participation in psychiatry placements change their perception? Furthermore, to what extent are psychiatry placements equipped to effectively deliver the new curriculum? Is there a preferred way to deliver psychiatric clinical teaching according to foundation doctors’ preferences? This study aims to answer these questions by providing insights from the Maudsley Training Programme in South London. To the best of our knowledge, this study is the first to address these questions and specifically evaluate the effectiveness of psychiatry placements in facilitating the attainment of the mental health competencies stipulated in the new UKFPC.

## Background

### Psychiatry clinical teaching in foundation training

There is a paucity of literature that examines the delivery of psychiatry curricula to foundation doctors or their perceptions on this topic. Since the implementation of the new curriculum, one study has highlighted the challenges faced in its delivery and proposed team-based learning facilitated by case vignettes as an effective method of delivery and improving confidence in skills.^5^ Another study proposed the use of didactic e-learning materials, including lectures and resources, to aid in the delivery of the new curriculum, with completion of the training rewarded with e-certificates;^6^ however, it is yet to be evaluated. Similarly, the literature on how psychiatry competencies are evaluated in foundation training is limited, highlighting an important gap.

### Psychiatry clinical teaching in other specialties

Expanding our search to include studies on teaching psychiatry competencies to other healthcare trainees has found that didactic approaches are associated with significant increases in recognition but not assessment and management skills.^7–10^ To address this, a combination of teaching methods such as didactic, experiential, problem- and case-based learning have been proposed.^11–13^ The preferred method of teaching evaluation was outcome analysis, which looked at evaluating Level 1 and 2 learning based on the Kirkpatrick evaluation framework,^14^ highlighting another important gap – the lack of Level 3 and 4 training programme evaluations.

## Method

### Study design

To evaluate the effectiveness of psychiatry clinical placements, this study employed Kirkpatrick's model of evaluation. The focus of the assessment was on measuring foundation doctors’ reactions to the training's perceived importance (Level 1) and their learning outcomes in terms of confidence gained (Level 2). A hybrid pre- and post-questionnaire was sent to all foundation doctors within the South London and Maudsley NHS Foundation Trust across three cohorts, totalling 135 foundation doctors, between August 2021 and March 2023. The questionnaire was administered before and after a 4-month psychiatric placement. Anonymised identifiers were used to match responses.

### Data collection

The pre- and post-questionnaires mainly comprised a core section of five anchor-point Likert scales. There are five Likert stem questions in total, of which three are composed of ten items reflecting the mandatory mental health conditions, and two are composed of seven items addressing clinical scenarios. The five-point Likert anchor values were codified as 1: ‘Not at all’; 2: ‘Slightly’; 3: ‘Fairly’; 4: ‘Very’; and 5: ‘Extremely’. The pre-questionnaire also included a multiple-choice question (MCQ) eliciting preferred teaching methods from the range offered in the placement, and two free-text questions to identify what foundation doctors would like to learn during their placements and obtain overall feedback on the placement.

### Data analysis

The ordinal data obtained from the Likert scale items was analysed using Microsoft Excel and IBM SPSS. Parametric *t*-tests were used to compare Likert scale means and measure whether the before and after results were significantly different; *t*-tests have previously been shown in simulation studies to work well with five-point Likert scales.^15,16^ We intended to use paired *t*-tests to analyse pre- and post-intervention measurements in this study. However, owing to a regrettable loss of data from cohort 3, a notable attrition rate and technical faults with the matching system, we decided to employ a conservative approach by adopting independent *t*-tests. This adjustment was deemed necessary in light of the data limitations. To address the issue of multiple hypothesis testing, Bonferroni correction was applied.

The qualitative data derived from the free-text questions proved insufficient for a comprehensive qualitative analysis owing to scarce and brief responses. Nevertheless, it provided valuable qualitative insights into specific areas of interest for foundation doctors during their psychiatry placement. To distil the essence of these insights, we organised the most recurrent patterns into concise codes.

## Ethics and consent

This study met the criteria for minimal risk research as defined by our institution's ethical guidelines and was conducted as part of the evaluation of a local training programme. Therefore, based on our institutional policies and guidelines, the project was registered as a minimal risk project on the King's College London REMAS system (project ID: 38756). Informed consent was obtained from all participants, and measures were taken to protect participant confidentiality and anonymity throughout the study.

This manuscript is an honest, accurate, and transparent account of the study being reported, and no important aspects of the study have been omitted.

## Results

### Data overview and response rate

Three cohorts of foundation doctors who had completed a 4-month psychiatry placement were administered a pre- and post-placement survey (*N* = 135). Regrettably, owing to technical issues, there was a loss of most quantitative data from cohort three; therefore, cohort three was excluded from the statistical analysis, leaving us with a total new sample of *N* = 90. Only the qualitative data and MCQ data were usable from all three cohorts (*n* = 63) and are described in the results section.

The overall pre-survey response rate was 63/135 (46.6%) for the free text boxes and MCQ, with a pre-survey response rate of 40/90 (44.4%) for the two cohorts included in the statistical analysis. The post-survey response rate from the two included cohorts was 24/90 (26.6%).

### Interpretation of results

The statistical analysis included data from a total of 40 foundation doctors in the pre-survey and 24 foundation doctors in the post-survey, with 14 foundation doctors overlapping and 26 (pre) and 10 (post) foundation doctors independent. Applying independent *t*-tests in the context of an overlap could lead to an artificial reduction in variance and an inflated *P*-value. This means that there might be a significant effect even if the result does not reach the statistical significance threshold. After applying Bonferroni correction, the thresholds for statistical significance in this study were *P* = 0.005 for perceived importance and confidence in recognising and assessing mental health conditions and *P* = 0.007 for perceived importance and confidence in managing clinical scenarios. Mode, median and mean values are included in Tables 1 and 2. The median was also utilised to visually represent the differences in Supplementary Figs. 1–5.

**Table 1:** Foundation doctors’ perceived importance of the curriculum pre- and post-placement

	Perceived importance						Independent-samples *t*-test			
	Pre-placement *N* = 40			Post-placement *N* = 24					CI	
	Mode	Median	Mean	Mode	Median	Mean	Mean difference	*P*-value	Lower	Upper
Mental health conditions										
Depression	5	5	4.53	5	5	4.72	−0.183	0.192	−0.461	0.094
Mania	4	4	3.97	5	5	4.40	−0.425	0.033	−0.815	−0.034
Psychosis	4	4	4.25	5	5	4.56	−0.310	0.071	−0.647	0.027
Anxiety/panic	5	4	4.27	5	5	4.36	−0.085	0.690	−0.509	0.339
Personality disorder	3	3	3.55	3, 4, 5	4	3.92	−0.370	0.114	−0.831	0.091
Delirium	5	5	4.67	5	5	4.72	−0.045	0.726	−0.300	0.210
Chronic cognitive impairment/dementia	4	4	4.4	5	5	4.56	−0.160	0.267	−0.445	0.125
Eating disorders	4	4	3.77	4	4	3.84	−0.065	0.757	−0.482	0.352
Substance use disorder	4	4	4.05	5	4	4.24	−0.190	0.344	−0.588	0.208
Somatisation disorders and functional syndromes	4	4	3.55	3	4	3.88	−0.330	0.191	−0.829	0.169
Clinical scenarios										
Assessing capacity and using Mental Capacity Act	5	5	4.6	5	5	4.44	0.160	0.290	−0.139	0.459
MHA 1983 including section 5(2)	4, 5	4	4.1	4	4	3.96	0.145	0.546	−0.332	0.623
Ethical framework around difficult decision-making	5	4	4.17	3, 4, 5	4	3.92	0.255	0.291	−0.223	0.733
Interplay of physical disease and psychiatric symptoms	5	5	4.45	4	4	4.32	0.130	0.424	−0.193	0.453
Serious adverse effects of psychopharmacology	5	5	4.6	4	4	4.20	0.400	0.025	0.050	0.749
Management of difficult patients at risk	4	4	4.47	4	4	4.20	0.275	0.060	−0.011	0.561
Explaining difficult diagnosis or of non-organic cause	4	4	4.07	4	4	3.84	0.238	0.300	−0.218	0.696

**Table 2:** Foundation doctors’ confidence in recognising, assessing and managing mental health competencies of the curriculum pre- and post-placement

	Confidence regarding mental health conditions and scenarios						Independent-samples *t*-test			
	Pre-placement *N* = 40			Post-placement *N* = 24					CI	
	Mode	Median	Mean	Mode	Median	Mean	Mean difference	*P*-value	Lower	Upper
Recognising mental health conditions										
Depression	3	3	3.46	4	4	4.28	−0.816	0.000	−1.147	−0.485
Mania	3	3	3.02	4	4	3.88	−0.855	0.000	−1.215	−0.494
Psychosis	3	3	3.25	4	4	4.08	−0.830	0.000	−1.222	−0.437
Anxiety/panic	4	4	3.46	4	4	3.88	−0.418	0.037	−0.810	−0.026
Personality disorder	2	2	2.17	3	3	3.28	−1.105	0.000	−1.553	−0.656
Delirium	4	4	3.6	4	4	3.84	−0.240	0.211	−0.619	0.139
Chronic cognitive impairment/dementia	3	3	3.42	4	4	3.48	−0.055	0.806	−0.499	0.389
Eating disorders	3	3	3.02	4	4	3.36	−0.335	0.220	−0.874	0.204
Substance use disorder	3	3	2.97	3	3	3.56	−0.585	0.012	−1.034	−0.135
Somatisation disorders and functional syndromes	1	2	1.95	3	3	3.00	−1.050	0.000	−1.546	−0.553
Assessing mental health conditions										
Depression	3	3	3.31	4	4	4.16	−0.842	0.000	−1.224	−0.461
Mania	3	3	2.56	4	4	3.64	−1.075	0.000	−1.504	−0.647
Psychosis	3	3	2.67	4	4	3.92	−1.245	0.000	−1.683	−0.806
Anxiety/panic	3	3	3.00	4	4	3.76	−0.760	0.001	−1.197	−0.322
Personality disorder	1	1	1.70	3	3	3.12	−1.420	0.000	−1.878	−0.961
Delirium	4	4	3.51	4	4	3.60	−0.087	0.664	−0.486	0.312
Chronic cognitive impairment/dementia	3	3	3.27	4	4	3.44	−0.165	0.462	−0.610	0.280
Eating disorders	3	2	2.35	3	3	2.92	−0.570	0.045	−1.125	−0.014
Substance use disorder	3	3	2.52	3	3	3.04	−0.515	0.031	−0.982	−0.047
Somatisation disorders and functional syndromes	1	1	1.65	3	3	2.88	−1.230	0.000	−1.682	−0.777
Managing clinical scenarios										
Assessing capacity and using Mental Capacity Act	3	3	3.17	4	4	3.92	−0.749	0.001	−1.195	−0.302
MHA 1983 including section 5(2)	2	2	2.22	3	3	3.12	−0.895	0.000	−1.365	−0.425
Ethical framework around difficult decision-making	2	2	2.17	3	3	3.12	−0.945	0.000	−1.409	−0.480
Interplay of physical disease and psychiatric symptoms	3	3	2.95	4	4	3.76	−0.810	0.000	−1.218	−0.401
Serious adverse effects of psychopharmacology	2	2	2.45	3	3	3.28	−0.830	0.000	−1.198	−0.461
Management of difficult patients at risk	3	3	2.70	4	4	3.68	−0.980	0.000	−1.433	−0.526
Explaining difficult diagnosis or of non-organic cause	2	2	2.52	4	3	3.36	−0.835	0.001	−1.314	−0.355

### Foundation doctors and perceived importance of the new psychiatry curriculum

Table 1 depicts the foundation doctors’ perceptions of the importance of being familiar with the new mental health competencies, as per Box 1, at their stage in training, pre- and post-placement. Initial ratings given by foundation doctors for perceived importance of the mental health competencies were positive across the board, with most respondents considering knowledge of the mental health competencies to be ‘Very’ or ‘Extremely’ important. Noticeable outliers were for personality Disorder, which showed the lowest valued responses, with a median and mode of 3 and a mean value of 3.55; and somatisation disorders and functional syndromes, which, although the median and mode would suggest that they were on par with the other highly rated competencies, showed a similarly low mean of 3.55. On the other hand, the competencies that were initially perceived as most important for the foundation doctors were depression, delirium, the Mental Health Act (MHA), and adverse effects of psychopharmacology, all with a mode and median of 5 and means of 4.53, 4.67, 4.6 and 4.6, respectively.

Owing to the relatively low number of responses and because initial perceived importance was already quite high, we did not see significant (*P* < 0.005) changes in perceived importance in most of the competencies. Although changes were not significant, a trend of slight increase in perceived importance across the board for all mental health conditions was observed, as well as a slight overall decrease in perceived importance of the clinical skills in key scenarios after the placement. The same data are visualised in Supplementary Figs. 1 and 2 with the use of diverging stacked bar charts.

### Foundation doctors’ confidence in applying psychiatric competencies to practice

Table 2 describes the confidence levels of foundation doctors in recognising, assessing and managing mental health conditions and clinical scenarios put forth by the new curriculum. Pre-placement, the foundation doctors’ confidence in recognising and assessing mental health conditions was medial, with most of them responding that they were ‘Fairly’ confident, whereas for clinical scenarios, they were mostly just ‘Slightly’ confident. Noteworthy outliers with respect to recognising mental health conditions were personality disorder and somatisation disorders, where we observed mean values of 2.17 and 1.95, respectively. This trend of low confidence in assessing these conditions continued post-placement, with mean values of 1.7 for personality disorder and only 1.65 for somatisation disorders.

Post-placement, there was a significant (*P* < 0.005; *P* < 0.007) increase in confidence for almost all measured competencies. The biggest improvements were again seen in the confidence of foundation doctors to recognise personality disorder, with a significant mean increase of 1.105 (*P* < 0.005), and somatisation disorders and functional syndromes, with a mean increase of 1.05 (*P* < 0.005). The increase in assessment confidence for these conditions was also among the most significant increases, with a mean difference of 1.42 (*P* < 0.005) for personality disorders and 1.23 (*P* < 0.005) for somatisation disorders and functional syndromes. Other positive outliers were seen for confidence in assessing mania and psychosis, with respective significant increases in the mean of 1.075 (*P* < 0.005) and 1.245 (*P* < 0.005). Confidence in managing clinical scenarios also showed significant increases (P < 0.007) across the board. There were no significant changes observed in foundation doctors’ confidence in recognising and assessing delirium, dementia, substance misuse disorder or eating disorders. Overall, we observed a strong pattern of improvement in foundation doctors’ confidence in recognising, assessing and managing curriculum-mandated competencies, with few exceptions. The same data are visualised in Supplementary Figs. 3–5 with the use of diverging stacked bar charts.

### Foundation doctors and their preferred learning method in achieving curricular competencies

**Fig. 1:**
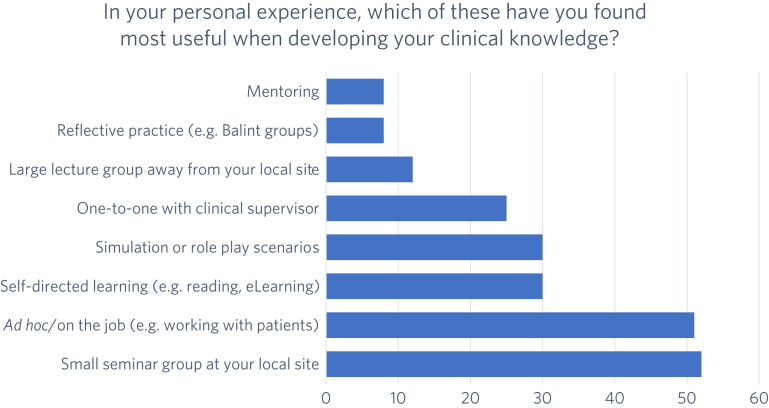
Preferred teaching method for achieving psychiatry competencies from the range offered in the placement (*N* = 63).

Of the 135 foundation doctors, 63 answered this question. The most favoured methods were small seminar groups at the doctor's local clinical site and *ad hoc* teaching on the job. The least favoured methods were mentoring, reflective practice and large lecture group teaching away from the local clinical site. These results are illustrated in Fig. 1.

### Foundation doctors and what they would have wanted to learn in their placements but did not

Twelve of 135 foundation doctors answered this question. Foundation doctors were asked what they would like to learn during their psychiatry placements, using a free-text box. We mapped the findings against the current curriculum to identify potential gaps between what foundation doctors want and what they receive. We have identified six areas of interest that were not captured in the new UKFPC, summarised below:

- emotional work of being a psychiatrist;
- communication skills training;
- learning about psychiatry career pathways;
- what academia looks like in psychiatry;
- experience of subspecialties, such as child and adolescent mental health services and learning disabilities;
- acute health context-based teaching.

## Discussion

In the UK, foundation training is the gateway between medical school and a medical career. Doctors are required to provide evidence of achievement of ‘foundation competence’ in order to progress to specialty training, in the form of a ‘Foundation Programme Certificate of Completion’ or ‘Certificate of Readiness to Enter Specialty Training’. Although this paper relates specifically to a UK Curriculum, we acknowledge parallel training programmes or medical internships across the globe, and we propose that foundations in psychiatric education are essential for all doctors as we face a ‘mental health pandemic’.

This study aimed to assess foundation doctors’ perceptions of the mental health competencies in the 2021 UKFPC. It also sought to determine whether confidence and perceived learning in these competencies improved after a 4-month psychiatry placement. In addition, it explored preferred methods of acquiring these competencies during the placement, and what foundation doctors might want to learn in a psychiatry placement beyond the curriculum.

The study found that after a 4-month psychiatry rotation, there was an overall improvement in the perceived importance and confidence levels of foundation doctors in recognising and assessing various psychiatric conditions. However, there was a decrease in the perceived importance of the skills and knowledge associated with the curricular clinical scenarios after the placement, indicating a potential failure in emphasising transferable skills and delivering acute health context-based teaching. The initial baseline confidence levels of foundation doctors were generally moderate, with an increase of 1 median point on average in confidence levels noted in most areas after the placement. This suggests that the psychiatric placement was successful in improving foundation doctors’ self-perceived confidence in their psychiatry curricular competency.

Foundation doctors on the Maudsley Training Programme complete a 4-month clinical placement in a psychiatric setting, complemented by monthly 4-hour psychiatry seminars for all foundation doctors, plus a variety of local and trust-wide educational opportunities. The seminars are designed to cover various topics of the curriculum based on group-identified preferences obtained from an initial survey and employ a range of teaching methods such as case-based discussions, problem-based learning and team-based learning. Our findings suggest that this combination of bedside teaching and clinical experience, traditional didactic lectures, and modern teaching strategies works well in improving group confidence overall. This is consistent with the existing literature, which suggests that using a combination of teaching methods is the most effective approach to medical education.^11–13^ Furthermore, it supports previous studies that have identified team-based learning as an effective standalone teaching method for foundation doctor psychiatry education.^5^ The scarce literature on effective strategies for delivering psychiatry competencies to foundation doctors highlights a critical gap in postgraduate medical education, particularly given the need for context-specific teaching in this field.

Mentoring and reflective practice were declared the least effective methods of developing clinical knowledge. There are no qualitative data available to help explain this, although we hypothesise that these two methods focus more on the emotional and problem-solving aspects of medical education than the acquisition of clinical knowledge. At the time of the study, the Maudsley Training Programme did not offer a formal mentoring scheme or reflective practice programme for foundation doctors. In the UK General Medical Council's latest *Good Medical Practice* guide,^17^ reflective practice and mentoring are explicitly referred to as prominent methods of ‘maintaining, developing and improving performance’, and so medical educators and future research should consider how best to introduce such opportunities for foundation doctors.

The training programme was evaluated using Kirkpatrick's evaluation model, focusing on levels 1 (reaction) and 2 (learning). The study assessed foundation doctors’ changes in attitude (confidence) after the psychiatric placement to evaluate their learning. Evaluating Kirkpatrick levels 3 (change in behaviour) and 4 (organisational performance) would require more intrusive and expansive tools, such as MCQs, Objective Structured Clinical Examination (OSCEs) or clinical key performance indicators. Given the low morale and high burnout rates at the time, in the context of the pandemic and industrial disputes,^18,19^ the authors were concerned that extra evaluation would be logistically challenging and poorly accepted. This highlights an important gap with respect to postgraduate medical education programmes in evaluating such programmes as a whole or various components holistically, specifically their impact on patient outcomes. Although less time- and resource-consuming methods could have been employed to measure at level 3, such as document analysis of the supervised learning events forms, the subjective nature of psychiatry competency, interrater and intrarater variability, as well as usually scarce and non-specific supervisor feedback practices, would affect the reliability and validity of the results. Further research is needed to improve both the quality of formative assessments and evaluative practice in postgraduate psychiatry.

This study had limitations that need to be considered. Owing to technical issues, the matching system did not work as expected; therefore, the study measured overall group confidence rather than individual confidence as initially planned. Moreover, owing to data loss, we were unable to link pre- and post-scores, so we had to use independent-sample *t*-tests as opposed to paired *t*-tests. This will have further lowered our power to detect a statistically significant result, with the consequence that the null findings may represent a type II error. Although the notable attrition rate is consistent with the literature on survey response, it is important to also acknowledge the potential for non-responder bias. Specifically, it is possible that foundation doctors who did not respond to the post-placement survey had different experiences or perceptions of the curriculum, which could have an impact on the generalisability of the findings, and therefore the results need to be interpreted cautiously and in context. Furthermore, this study measured perceived competence rather than objective change in behaviour, knowledge or skill acquisition, and this may be an inaccurate predictor of the acquisition of clinical skills.^20,21^ It is possible that foundation doctors may have over- or underestimated their level of learning and confidence. Finally, our study was limited to the perspective of foundation doctors on the Maudsley Training Programme, a metropolitan psychiatry training scheme that has strong links with an international psychiatry research institute, The Institute of Psychiatry, Psychology and Neuroscience. Therefore, our results cannot be extrapolated to represent the national foundation doctor cohort. In conclusion, this study's limitations affect the generalisability of the results, and all information needs to be interpreted in context.

Despite the limitations of the study, this paper underscores a previously overlooked facet within medical education research – the evaluation of psychiatry placements in instilling mental health competencies in non-specialised foundation doctors – and it highlights another gap in the research on efficacious teaching strategies for cultivating transferable psychiatry skills to other settings such as acute medicine and general practice. These settings are fundamentally different from psychiatry in terms of resources such as time and infrastructure in place. Further research is needed to understand the current state of postgraduate medical education in psychiatry with respect to facilitating mandatory competency across all specialties and grades, including general practice and psychiatry trainees. Mixed and qualitative study designs should be employed to better understand the educational values of training placements and the learner's needs. Future studies could explore whether there is any correlation between prior exposure to psychiatry during the undergraduate years and increased confidence in recognising, assessing and managing mental health disorders at a postgraduate level.

Context teaching in healthcare refers to the use of real-world contextual scenarios in teaching knowledge and skills. This type of teaching aims to bridge the gap between theory and practice by providing learners with the opportunity to apply knowledge in a real-world setting that closely resembles their clinical environment. In the case of foundation doctors, the clinical environment is the acute health setting. Examples of context teaching are risk assessment in an accident and emergency setting, recognition and assessment of acute organic psychosis, use of capacity and the MHA in the acute hospital, etc., where resources such as time and space differ greatly from those of psychiatry settings. Providing learners with opportunities to apply their knowledge in a contextualised setting makes them better able to understand and integrate the information into their practice. This type of teaching also helps learners to develop critical thinking, problem-solving and decision-making skills that are essential in healthcare practice.

A final urgent question is: how can foundation doctors attain mental health competencies in the new UKFPC if they are not assigned to a psychiatry placement? Foundation training programme directors need to consider how foundation doctors are exposed to clinical scenarios and teaching environments that enable adequate psychiatric learning. This raises the question of inequity of access to training opportunities across the country and risks an uneven distribution of psychiatric competency in tomorrow's doctors and disparities in future patient care. Moving forward, it will be essential to address these issues to ensure that doctors in training receive a high-quality and consistent education and are well prepared to provide quality care to patients with mental health needs. A potential solution could be an online e-learning environment, as proposed by Lewis et al, pending their evaluative results.^5^

## Conclusions

This study assessed foundation doctors’ perceptions of the mental health competencies in the 2021 UKFPC and determined whether confidence and perceived learning in these competencies improved after a 4-month psychiatry placement. The study found that although there was an overall improvement in foundation doctors’ perceived importance and confidence levels in recognising and assessing various psychiatric conditions after the placement, there was a decrease in the perceived importance of the skills and knowledge associated with the curricular clinical scenarios, indicating a potential failure in emphasising transferable skills and delivering context-specific teaching. The discussion presented here highlights the importance of context teaching and constructive alignment in medical education, identifies gaps in postgraduate medical education, and raises ethical concerns related to access to training opportunities, variation in training quality, and assessment and evaluation of learning outcomes. Moving forward, efforts should be made to create effective curriculum delivery and assessment strategies to improve the quality and relevance of medical education for foundation doctors and other healthcare professionals.

## Supporting information

Varvari et al. supplementary material Varvari et al. supplementary material

## Data Availability

The data that support the findings of this study are available on request from the corresponding author.

## About the authors

**Ioana Varvari** is a Clinical Fellow in Treatment Resistant Schizophrenia at Oxford Health NHS Trust, Oxford, UK. **Thomas Dewhurst** is a consultant psychiatrist at South London and Maudsley Mental Health NHS Trust, London, UK. **Corinne Jones** is the Postgraduate Psychiatry Medical Education Manager at South London and Maudsley Mental Health NHS Trust, London, UK. **Richard Haslam** is a consultant psychiatrist and foundation doctor tutor at South London and Maudsley Mental Health NHS Trust, London, UK.
