# Supplementary material for: The new Foundation Programme Mental Health Curriculum: foundation doctors’ perceptions of its importance and their competency: pre–post psychiatric placement evaluation study
Source: BJPsych Bull. 2025 Jun;49(3):192–9. doi: 10.1192/bjb.2024.18 (PMC12171842; doi:10.1192/bjb.2024.18)
Supplement: Varvari et al. supplementary material [file S2056469424000184sup001.docx]

The New Foundation Programme Mental Health Curriculum: Foundation Doctors' Perception of Its Importance and Their Competency. A Pre and Post Psychiatric Placement Evaluation Study

**- ONLINE SUPPLEMENT –**

**
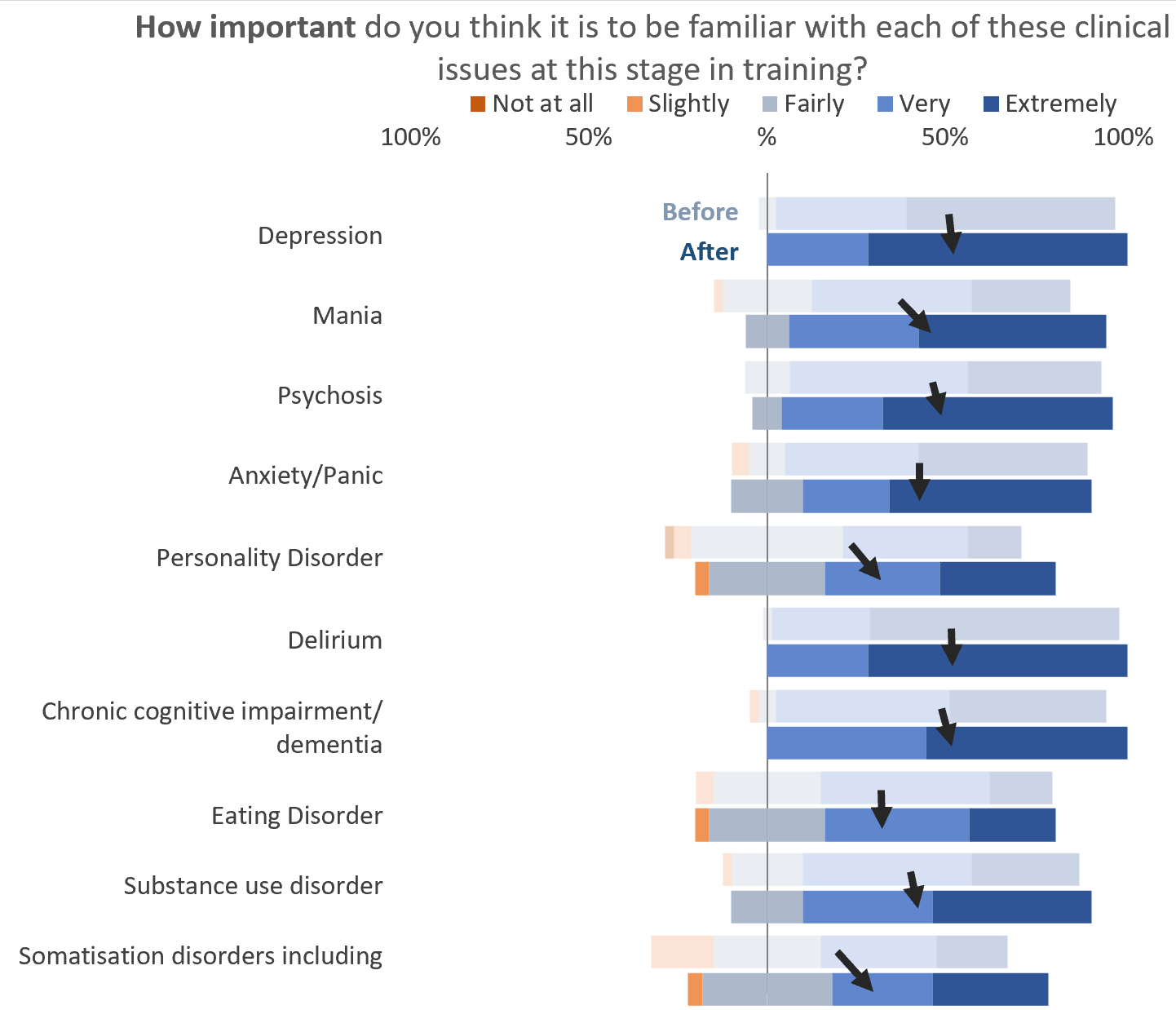
**

**Figure 1.** Visualisation of the responses and FDs’ level of perceived importance for conditions outlined by the curriculum after a 4-month psychiatry placement on both cohorts combined. (The arrows start from and point to the median of each before and after section and show the magnitude of change (length of the arrow) between the responses. To the right of the medial line, in blue, are considered desirable responses, and to the left, undesirable responses in orange.)

**
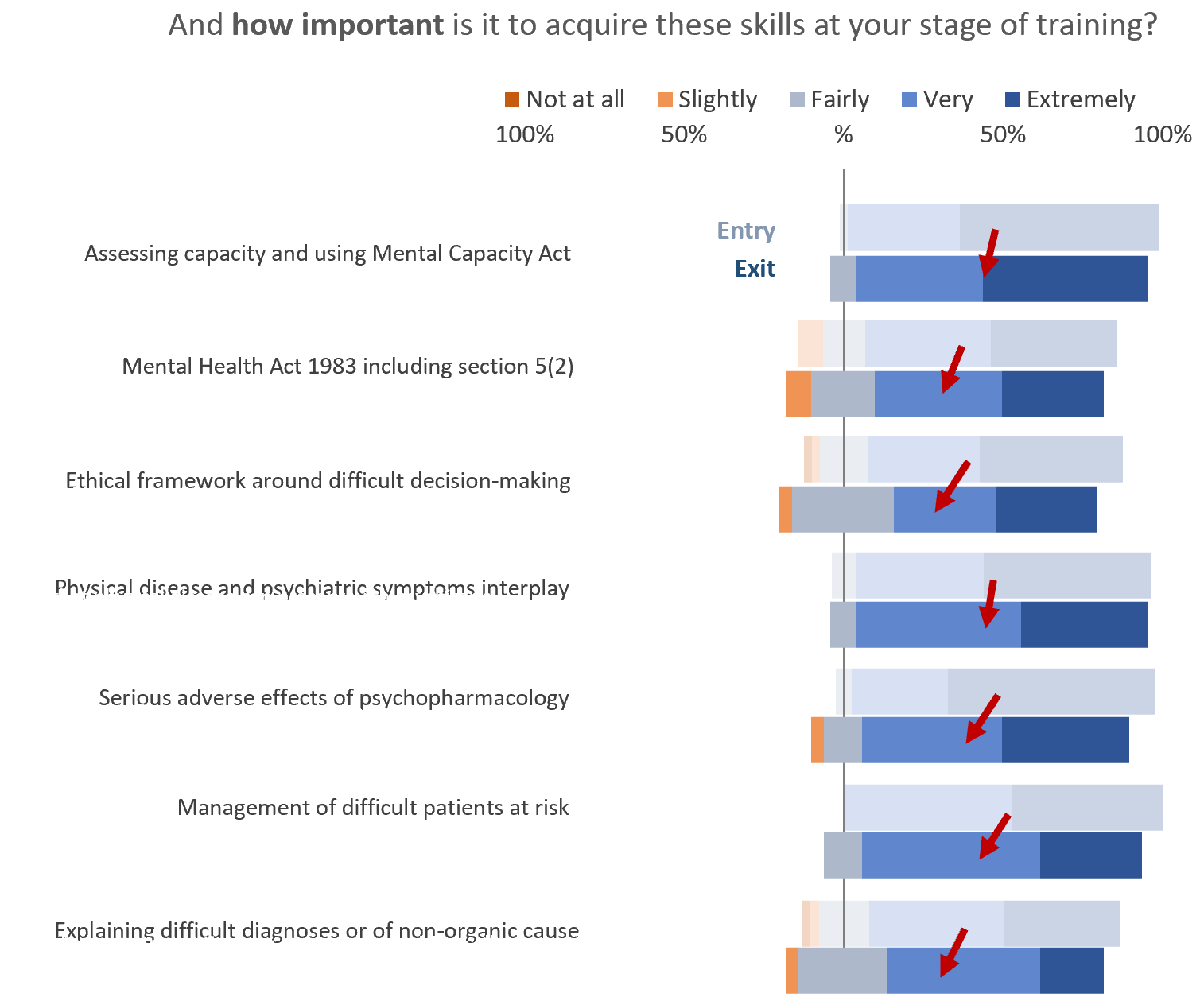
**

**Figure 2.** Visualisation of the responses and FDs’ level of perceived importance for conditions outlined by the curriculum after a 4-month psychiatry placement on both cohorts combined. (The arrows start from and point to the median of each before and after section and show the magnitude of change (length of the arrow) between the responses. To the right of the medial line, in blue, are considered desirable responses, and to the left, undesirable responses in orange.)


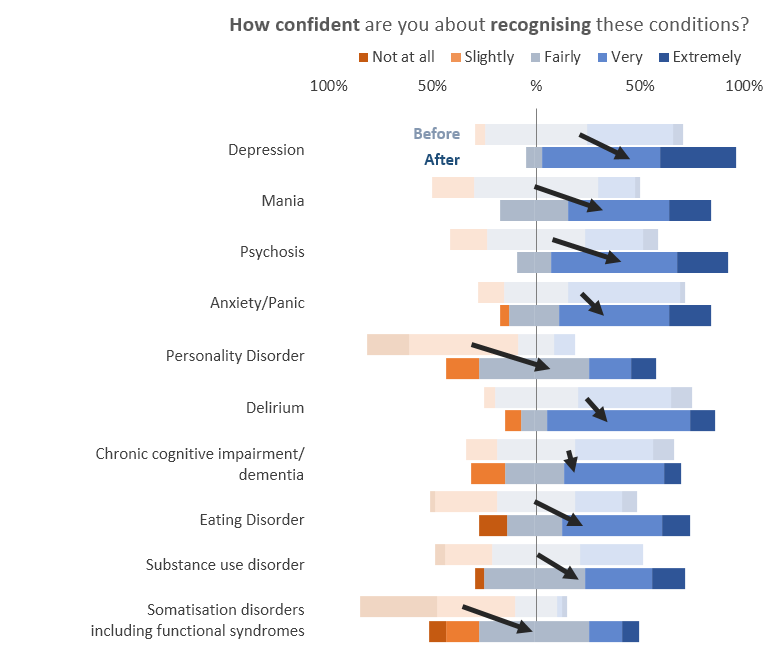


**Figure 3.** Visualisation of the responses and confidence level change in **RECOGNISING** conditions outlined by the curriculum after a 4-month psychiatry placement on both cohorts combined. (The arrows start from and point to the median of each before and after section and show the magnitude of change (length of the arrow) between the responses. To the right of the medial line, in blue, are considered desirable responses, and to the left, undesirable responses in orange.)


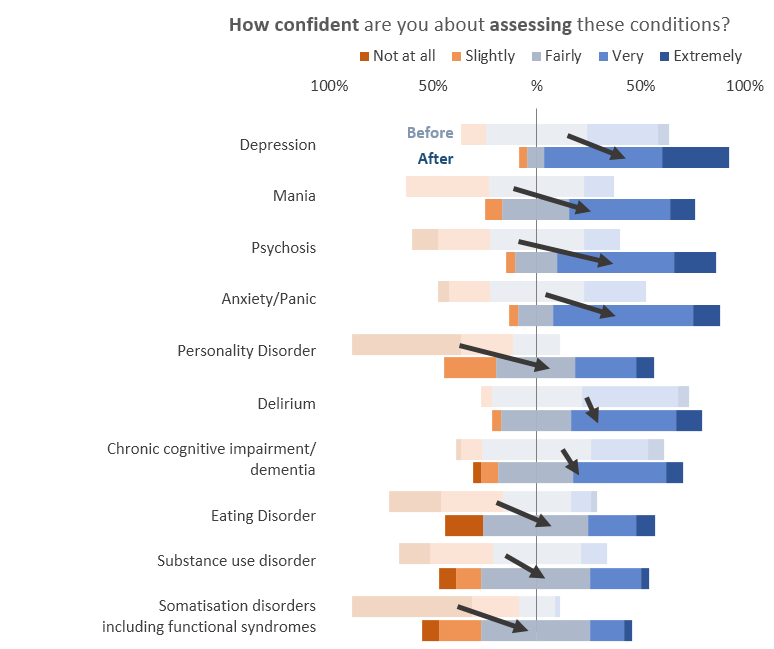


**Figure 4.** Visualisation of the responses and confidence level change in **ASSESSING** conditions outlined by the curriculum after a 4-month psychiatry placement on both cohorts combined. (The arrows start from and point to the median of each before and after section and show the magnitude of change (length of the arrow) between the responses. To the right of the medial line, in blue, are considered desirable responses, and to the left, undesirable responses in orange.)


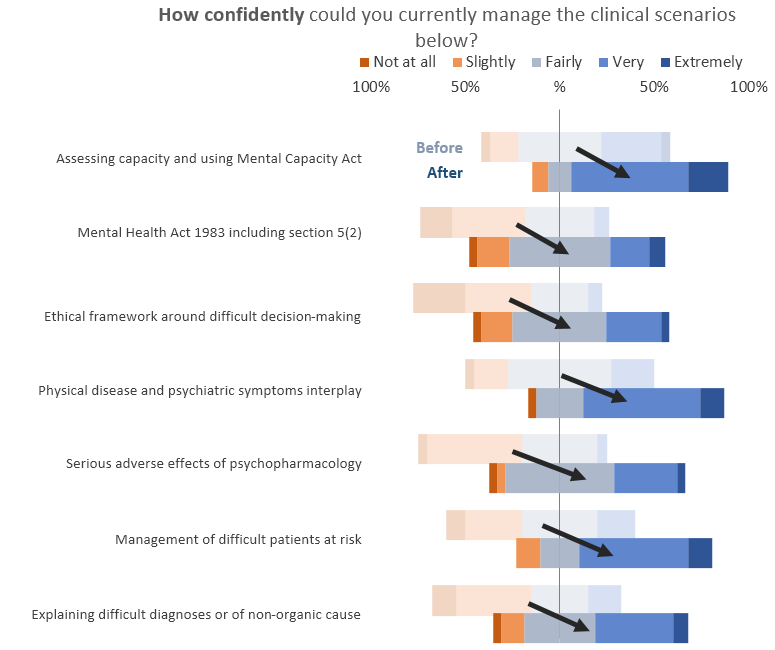


**Figure 5.** Visualisation of the responses and confidence level change in **MANAGING** conditions outlined by the curriculum after a 4-month psychiatry placement on both cohorts combined. (The arrows start from and point to the median of each before and after section and show the magnitude of change (length of the arrow) between the responses. To the right of the medial line, in blue, are considered desirable responses, and to the left, undesirable responses in orange.)
